# Supplementary material for: Evaluating Therapeutic Efficacy of the Vascular Disrupting Agent OXi8007 Against Kidney Cancer in Mice
Source: Cancers (Basel). 2025 Feb 24;17(5):771. doi: 10.3390/cancers17050771 (PMC11898701; doi:10.3390/cancers17050771)
Supplement: Supplementary file 1 [file cancers-17-00771-s001.zip › cancers-3467412-supplementary.pdf]

Supplementary Materials

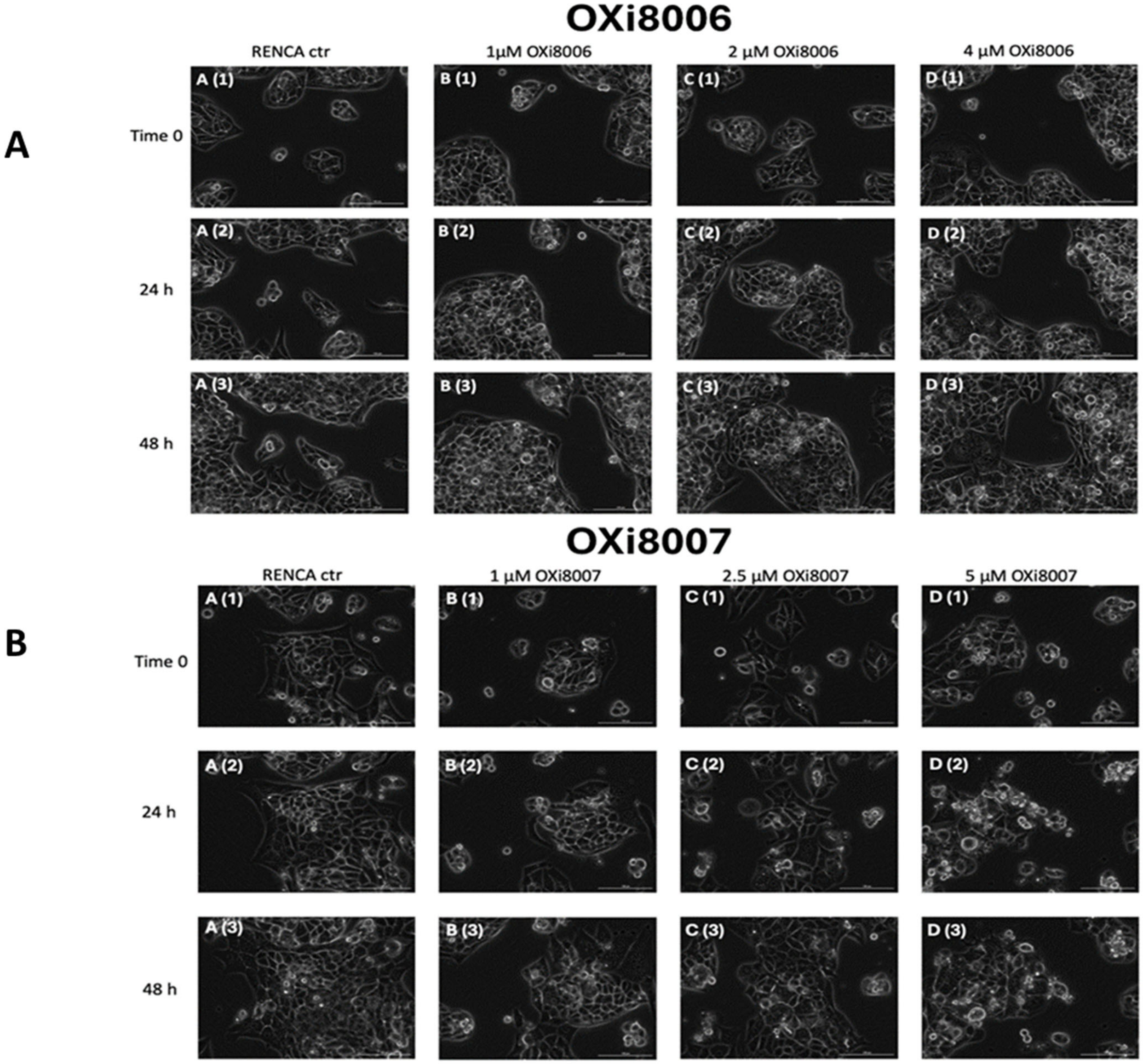

Figure S1. Renca cell morphology following treatment with OXi8006 or OXi8007. Photomicrographs show plates of Renca cells at various times following exposure to increasing concentrations of **A)** OXi8006 or **B**) OXi8007.

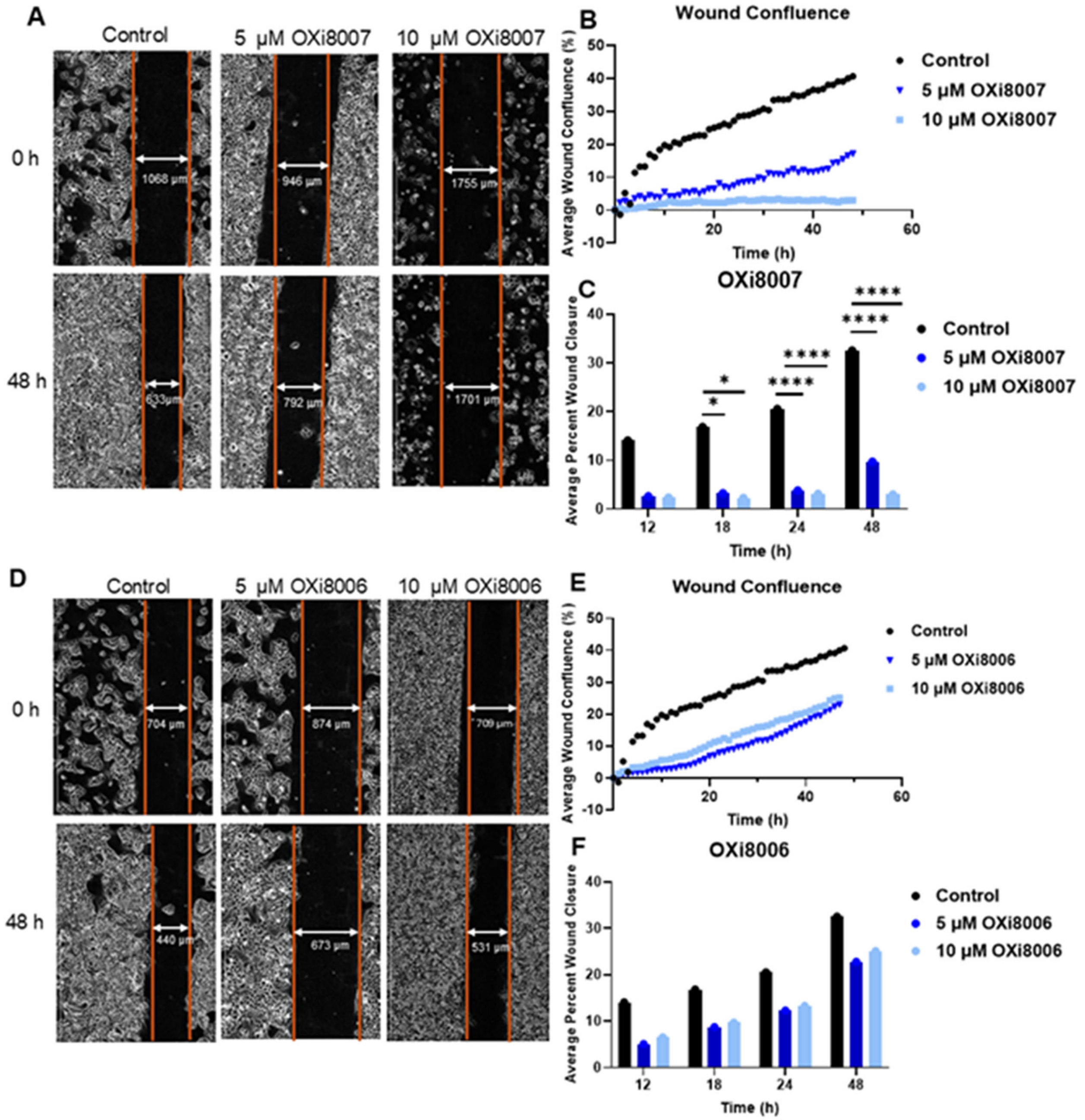

Figure S2- Wound Healing Assay of RENCA cells Comparison of control cells and cells treated with 5 μM or 10 μM OXi8006 and OXi8007. The RENCA cells migrated slowly into the scratch area, but proliferated rapidly growing on top of other cells that spilled over into the scratch area for an approximate closure of 33% at 48 h. Cells treated with OXi8006 demonstrated significantly reduced migration relative to control cells at early time points, but recovered and started to proliferate rapidly at later time points. Cells treated with OXi8007 showed significantly reduced migration relative to control cells at early time points, and decreased proliferation and cell death (10 μM) at later times. Vertical lines show cell-free wound area and wound width.

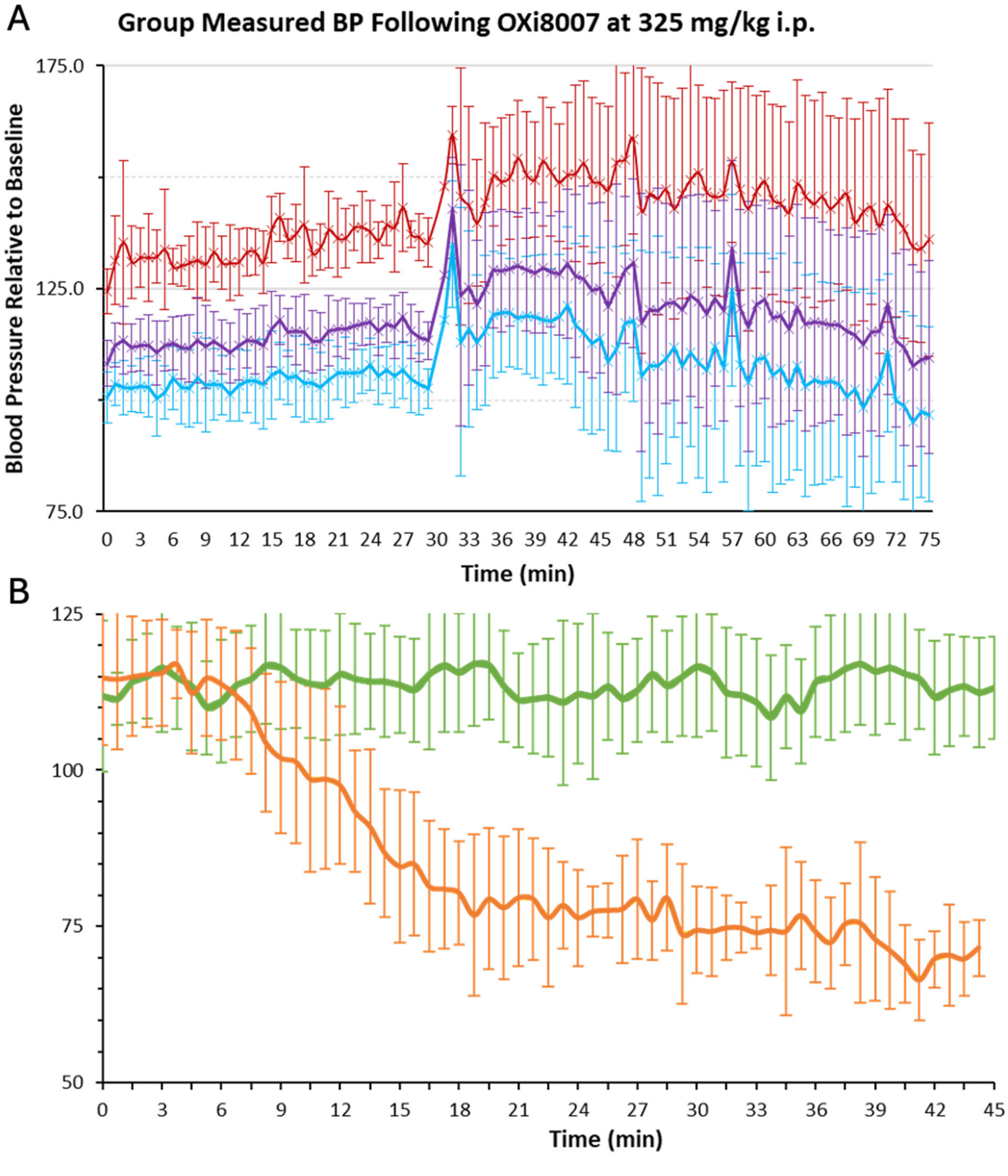

Figure S3 Blood pressure measurements in response to intervention. A) Systolic (red), diastolic (blue) and mean (purple) blood pressure with respect to administering 325 mg/kg OXi8007. **B)** Mean blood pressure response in a group of mice (n=5) to administration of saline (100 µl, IP) or hydralazine (1 mg/kg, IP). The significant response to HDZ verifies the ability to observe changes in BP in response to an intervention.

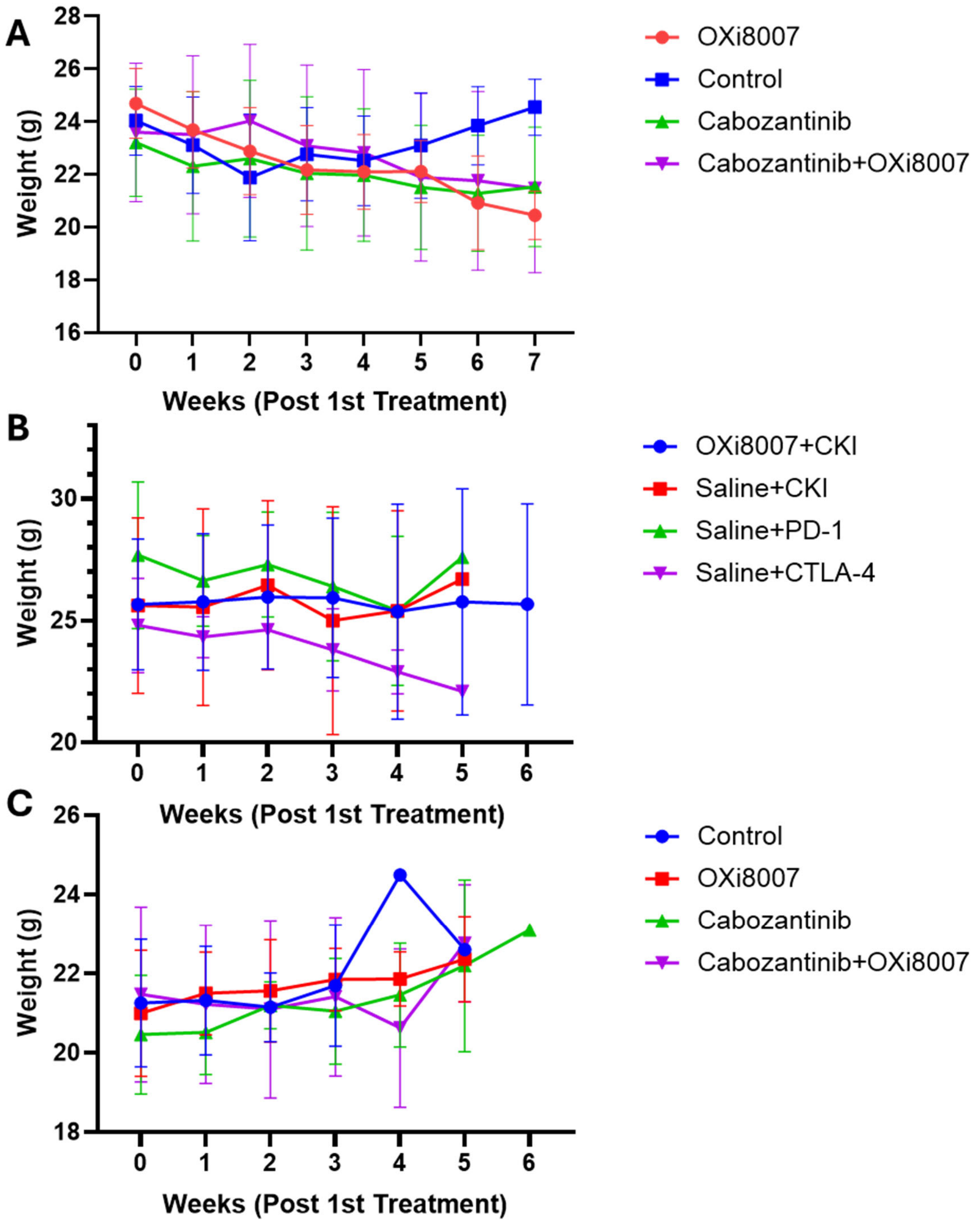

Figure S4. Weight fluctuations for treatment groups. A) Renca-luc tumor bearing BALB/c mice treated with OXi8007 and or cabozantinib. Treatment with cabozantinib alone (3 mg/kg daily) caused significant weight loss over the course of treatment compared with untreated control mice. ($p<0.005$). OXi8007 alone or in combination did not cause any significant body weight changes over the 7 weeks compared. Comparing body weight at week 7 versus baseline there was a significant loss in weight for animals treated with OXi8007 alone ($p = 0.0007$), B) Renca-luc tumor bearing BALB/c mice treated with OXi8007 and or check point inhibitors. Treatment with Anti-CTLA caused significant body weight loss compared with each of the other treatment groups by week three, C) No significant body weight changes were observed for any of the groups NOD/SCID mice bearing XP 258 tumors treated with OXi8007 and or cabozantinib.
